# Blood pressure status and recurrence risk after endovascular treatment of intracranial aneurysms: a systematic review and meta-analysis with narrative synthesis of blood pressure targets and hemodynamic evidence

**DOI:** 10.3389/fneur.2026.1928054

**Published:** 2026-10-26

**Authors:** Weiyuan Pan, Yu Xu, Heng Gao

**Affiliations:** Jiangyin Clinical College, Xuzhou Medical University, Jiangyin, Jiangsu, China

**Keywords:** blood pressure variability, computational fluid dynamics, endovascular coiling, hypertension, intracranial aneurysm, recanalization, recurrence, reperfusion

## Abstract

**Background:**

Recurrence, recanalization, reperfusion, or retreatment remains an important durability limitation after endovascular treatment of intracranial aneurysms. Most recurrence studies characterize blood pressure exposure as a history or study-defined diagnosis of hypertension rather than achieved post-treatment systolic or diastolic blood pressure.

**Objective:**

To quantify the association between study-defined/history-based hypertension status and recurrence-related outcomes after endovascular aneurysm treatment, and to separately summarize indirect clinical evidence on achieved blood pressure, blood pressure variability, and mechanistic hemodynamic evidence.

**Methods:**

PubMed/MEDLINE, Ovid MEDLINE, and Embase were searched from database inception through April 2026. The direct recurrence synthesis included adult endovascular aneurysm cohorts reporting recurrence-related outcomes according to hypertension status. Indirect clinical studies of achieved blood pressure or blood pressure variability and mechanistic computational-fluid-dynamics studies were synthesized separately. Odds ratios (ORs) for the direct recurrence analysis were pooled using inverse-variance random-effects models with restricted maximum-likelihood estimation and Hartung-Knapp adjustment. Study selection was independently performed by two reviewers at title/abstract and full-text stages; unresolved disagreements were adjudicated by a third reviewer.

**Results:**

Thirty-one studies were included. Eight studies provided extractable 2 × 2 data for the primary recurrence-related analysis (2,442 observations: 1,231 in the hypertension/high-BP-status group and 1,211 in the non-hypertension group; 559 recurrence-related events overall, 242 and 317 respectively). Study-defined hypertension showed a directionally higher but statistically non-significant association with recurrence/recanalization/reperfusion (OR 1.36, 95% CI 0.92–2.00; *p* = 0.104; *I*^2^ = 31.4%). No eligible recurrence study reported recurrence according to achieved SBP/DBP target categories. The overlapping full and matched Lee 2021 controlled-versus-uncontrolled hypertension estimates were presented separately and were not pooled. BP-variability studies addressed heterogeneous clinical outcomes and were not quantitatively pooled.

**Conclusion:**

Current evidence is insufficient to establish study-defined hypertension as an independent predictor of recurrence after endovascular aneurysm treatment or to recommend a recurrence-preventive blood pressure target. The available direct evidence concerns predominantly history-based hypertension status, whereas achieved blood pressure and blood pressure variability evidence is indirect for angiographic recurrence.

## Introduction

Endovascular treatment has become a major therapeutic strategy for ruptured and unruptured intracranial aneurysms, particularly after randomized and long-term comparative evidence demonstrated the clinical role of coiling in suitable ruptured aneurysms (1, 2). Although coiling, balloon-assisted coiling, stent-assisted coiling, flow diversion, and related embolization techniques reduce procedural invasiveness in selected patients, durability remains a central limitation. Recanalization, reperfusion, reopening, and retreatment after embolization are repeatedly reported across prospective cohorts, long-term observational series, and systematic reviews, with recurrence risk influenced by aneurysm, treatment, and follow-up characteristics (3–8).

Established recurrence predictors include larger aneurysm size, wide neck, rupture status, incomplete initial occlusion, Raymond-Roy occlusion grade, low packing density, residual aneurysm volume, parent-artery geometry, and treatment technique (3, 8–15). By contrast, the contribution of systemic BP exposure is less certain. Hypertension is biologically plausible as a recurrence-related factor because it contributes to vascular inflammation, wall remodeling, aneurysm growth, and rupture risk; however, most recurrence studies report only a history of hypertension, not longitudinal achieved BP values after embolization (16–18).

This distinction is central to interpretation. Study-defined hypertension generally reflects a baseline diagnosis, medical history, antihypertensive treatment, or threshold-based classification and is not equivalent to achieved post-treatment SBP, DBP, or MAP. A blood pressure target requires longitudinal exposure data linked directly to recurrence outcomes. Lee et al. evaluated systemic hypertension and controlled versus uncontrolled hypertension after coiling, whereas the other primary recurrence cohorts largely treated hypertension as a baseline covariate. No eligible recurrence study reported recurrence outcomes according to achieved SBP/DBP target categories (9–12, 14, 16, 19, 20).

Local hemodynamic evidence provides mechanistic context but is distinct from systemic blood pressure exposure. Computational fluid dynamics studies have associated recurrence with persistent wall shear stress, high flow velocity, pressure gradients at the coil-mass surface, residual flow volume, and complex post-embolization flow patterns (11, 21–31). These findings were treated as mechanistic evidence rather than as direct evidence that a specific systemic BP target prevents recurrence. The primary objective was the association between study-defined hypertension status and recurrence-related outcomes; achieved-BP/BP-variability and CFD evidence were complementary secondary evidence streams.

## Methods

### Design and reporting

This systematic review and meta-analysis study was conducted according to PRISMA principles. The review protocol was not prospectively registered. Evidence was organized into three analytically distinct streams: direct recurrence evidence, indirect clinical BP/BP-variability evidence, and mechanistic CFD/hemodynamic evidence. This separation was maintained in eligibility assessment, extraction, synthesis, tables, figures, and interpretation.

PICOS framework. Direct recurrence evidence: Population, adults with intracranial aneurysms treated endovascularly; Exposure, study-defined/history-based hypertension or hypertension-control status; Comparator, non-hypertension or controlled hypertension; Outcomes, angiographic recurrence, recanalization, reperfusion, reopening, or recurrence-related retreatment; Study designs, original observational cohorts with extractable exposure-outcome data. Indirect clinical BP evidence included achieved SBP/DBP, BP categories, or BP-variability metrics linked to functional outcome, mortality, rebleeding, or composite vascular prognosis and was not pooled with recurrence. Mechanistic evidence included patient-specific CFD or related hemodynamic measures linked to recurrence/recanalization and was interpreted only as biological plausibility, not as systemic BP-target evidence.

### Search strategy and study selection

PubMed/MEDLINE, Ovid MEDLINE, and Embase were searched from database inception through April 2026 using controlled vocabulary and free-text terms for intracranial aneurysm, endovascular treatment or embolization, recurrence/recanalization/reperfusion/retreatment, and BP/hypertension/BP variability. A supplementary broad recurrence search without mandatory BP terms was used because hypertension was frequently reported only in baseline or prognostic tables. The complete strategies are provided in Supplementary Table S1. After duplicate removal, title and abstract screening and subsequent full-text eligibility assessment were performed independently by two reviewers against the prespecified eligibility criteria. Disagreements regarding study eligibility were initially discussed between the two reviewers; when consensus could not be reached, a third reviewer independently adjudicated the disagreement and determined final eligibility.

### Eligibility criteria

#### Inclusion criteria

Studies were eligible for inclusion if they met all of the following criteria: (1) original human clinical studies including adult patients with ruptured or unruptured intracranial aneurysms; (2) aneurysms treated by an endovascular approach, including coiling, balloon-assisted coiling, stent-assisted coiling, flow diversion, or other endovascular embolization techniques; (3) reporting at least one recurrence-related outcome, including angiographic recurrence, recanalization, reperfusion, reopening, occlusion worsening, retreatment, repeated recurrence, rebleeding, or relevant post-treatment clinical prognosis; and (4) reporting at least one BP-related, hypertension-related, BP-control, BP-variability, or hemodynamic predictor. Randomized trials, prospective cohort studies, retrospective cohort studies, registry-based studies, case–control studies, and clinically relevant computational fluid dynamics studies were considered eligible. Studies were included in the quantitative meta-analysis only when recurrence-related data by hypertension/high-BP status or a relevant hypertension-related effect estimate were extractable. Studies without extractable recurrence-by-BP data were retained for narrative synthesis if they provided relevant BP-target, BP-variability, clinical-outcome, or hemodynamic evidence.

#### Exclusion criteria

Studies were excluded if they were reviews, editorials, letters without original data, conference abstracts without sufficient extractable information, animal or experimental-only studies, pediatric-only studies, surgical clipping-only studies, case reports, very small case series without analyzable outcomes, protocol or pre-results reports, or duplicate reports of the same population without additional relevant data. Studies were excluded from the quantitative recurrence meta-analysis if they did not provide extractable hypertension/high-BP status recurrence data, did not report a relevant BP-related effect estimate, or assessed outcomes that were not compatible with angiographic recurrence or recanalization. Studies meeting the broader systematic review criteria but lacking quantitative recurrence-by-BP data were retained only for narrative synthesis when they addressed indirect evidence relevant to BP targets, BP variability, rebleeding, clinical prognosis, or local hemodynamic recurrence mechanisms.

### Exposure and outcome definitions

The primary exposure was study-defined/history-based hypertension status. When a study explicitly defined hypertension, that definition was retained; otherwise hypertension was classified as reported by the source study and its operational definition was recorded as not reported. Achieved SBP/DBP or MAP values, target categories, and BP-variability indices were considered distinct exposures and were not interpreted as equivalent to hypertension history. The primary outcome domain comprised angiographic recurrence, recanalization, or reperfusion after endovascular treatment. Retreatment and repeated recurrence were analyzed only in supportive or sensitivity contexts. Functional outcome, mortality, acute rebleeding, and composite vascular prognosis were classified as indirect clinical outcomes.

### Data extraction and evidence classification

Data extraction was performed independently by two reviewers using a standardized extraction framework. Extracted variables included author, year, country, study design, sample size, analytical unit (patient or aneurysm), endovascular technique, follow-up duration, hypertension or BP definition, recurrence-related outcome definition, event counts and denominators, and reported adjusted estimates when available. For studies contributing to the quantitative synthesis, the four cells of each hypertension-versus-non-hypertension 2 × 2 table were independently extracted and verified against the corresponding source report before analysis. Discrepancies in extracted data were resolved by re-examination of the full-text source and retention of the source-supported value. Direct recurrence evidence, indirect clinical BP/BP-variability evidence, and mechanistic CFD evidence were extracted and synthesized separately.

### Risk-of-bias assessment

Risk of bias was assessed independently by two reviewers using the Quality In Prognosis Studies (QUIPS) framework. The six domains evaluated were study participation, study attrition, prognostic-factor measurement, outcome measurement, study confounding, and statistical analysis/reporting. Each domain was judged as having low, moderate, or high risk of bias. Disagreements between the two reviewers were resolved through discussion and consensus. No numerical quality score or summed quality category was calculated. Supplementary Table S2 reports the domain-level QUIPS judgments, and Supplementary Table S3 summarizes each study’s overall risk-of-bias interpretation and principal limitations.

### Statistical analysis

Odds ratios were pooled using inverse-variance random-effects models with restricted maximum-likelihood estimation of between-study variance and Hartung-Knapp adjustment. Heterogeneity was quantified using Cochran *Q*, tau^2^, tau, and *I*^2^. The primary model included one extractable study-defined hypertension-versus-non-hypertension comparison per study for recurrence/recanalization/reperfusion. Sensitivity analyses used a strict angiographic recurrence/recanalization definition, replaced the matched Lee 2021 cohort with its full cohort, added Han 2022 repeated-recurrence data, and excluded location-specific CFD cohorts. The full and matched Lee 2021 uncontrolled-versus-controlled hypertension estimates were not pooled because the cohorts overlap. BP/BP-variability estimates with different exposure scales and non-recurrence clinical outcomes were presented individually and not pooled. Supportive hypertension-related estimates reported by studies with differing outcome definitions or adjustment strategies were likewise displayed individually without a pooled summary. Funnel plots and leave-one-out analyses were interpreted cautiously because the quantitative models contained fewer than ten studies. Analyses were performed using RStudio (2026.01.2 Build 418).

## Results

### Study selection and evidence classification

The database search identified 1,219 records. After removal of 583 duplicates, 636 records were screened by title and abstract, of which 604 were excluded. Thirty-two full-text reports were assessed for eligibility; one protocol/pre-results report was excluded because it did not provide analyzable outcome data. Thirty-one studies were included in the systematic review. Eleven distinct studies contributed to at least one quantitative or supportive synthesis, including eight studies in the primary hypertension/high-BP status meta-analysis and three additional studies used only in sensitivity or supportive analyses. Twenty studies were retained for qualitative or narrative synthesis only, including six BP-target/BP-variability clinical-outcome studies, seven hemodynamic/CFD-only recurrence studies, and seven other recurrence or retreatment predictor studies (Figure 1).

**Figure 1:**
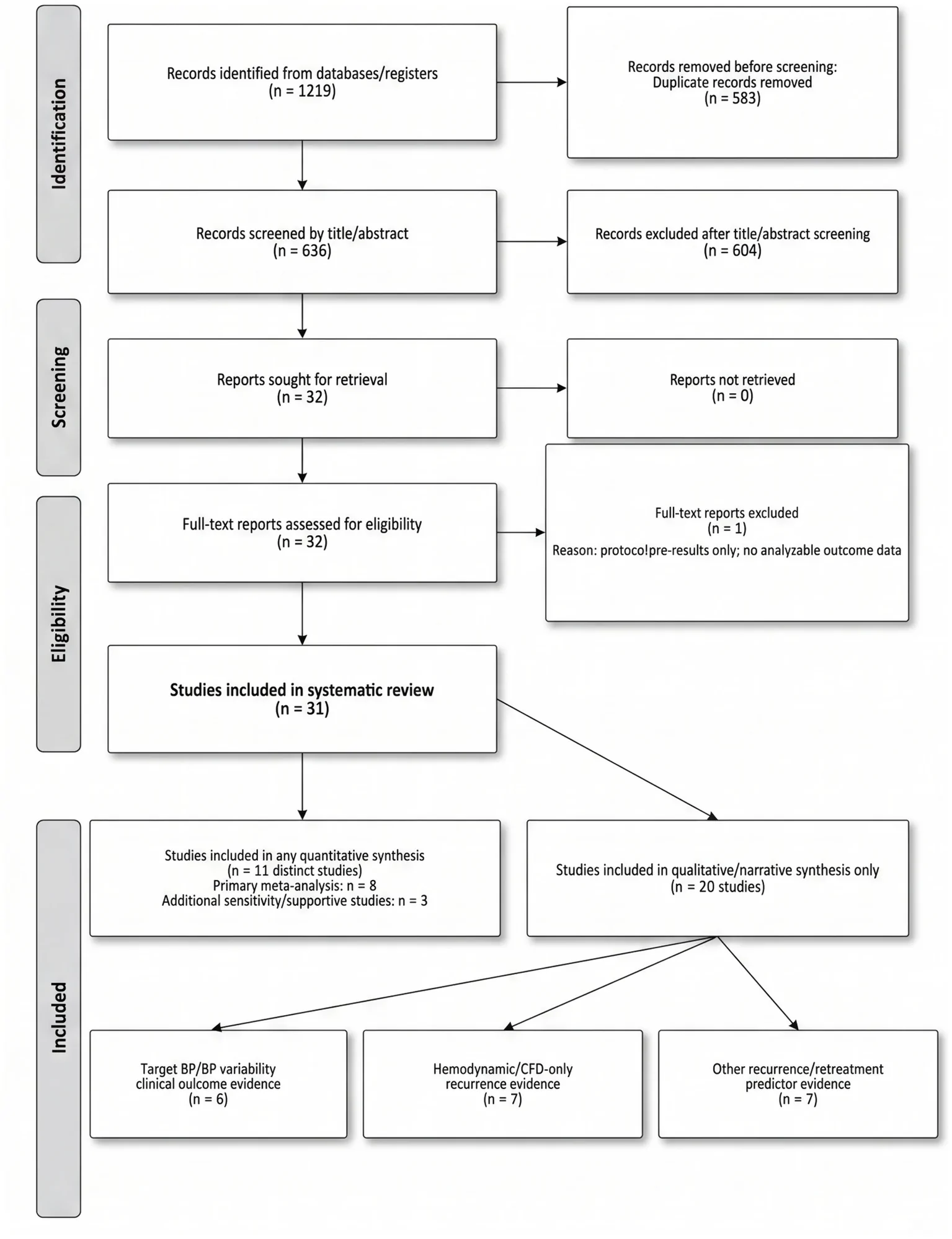
PRISMA flow diagram for inclusion of studies.

### Characteristics of included studies

The primary quantitative analysis included Lee 2021, Huang 2017, He 2023, Andereggen 2024, Sheng 2019, Yuan 2021, Zhang 2018, and Won 2024. Table 1 provides study-level comparability data for these eight studies, including sample size, patient-versus-aneurysm analytical unit, follow-up, hypertension definition, recurrence-related definition, and treatment characteristics. The studies differed materially in these domains, which informed the sensitivity analyses and the interpretation of indirectness (Table 2).

**Table 1:** Comparability of the eight studies included in the primary hypertension-status meta-analysis.

Study; sample; analytical unit	Follow-up	Hypertension definition	Recurrence-related definition	Relevant treatment characteristics	Analytical role
Lee 2021 (16); 715 coiled aneurysms; aneurysm-level outcome	Mean 28.6 ± 9.7 months; imaging at 6, 12, 24, and 36 months	Ongoing treatment for diagnosed hypertension or post-coiling SBP > 140 mmHg or DBP > 90 mmHg on >2 consecutive outpatient visits	Raymond-based minor or major recanalization during follow-up	Coil embolization; matched HTN/non-HTN analysis; stent use permitted	Matched comparison used in primary model
Huang 2017 (9); 658 patients with completely embolized saccular aneurysms; patient-level	>6 months; follow-up categorized as ≤6, 6–12, 12–24, and >24 months	History/clinical diagnosis reported; operational BP threshold not specified	Loosening/compression of coil or new contrast filling in aneurysm body/neck versus immediate post-procedure angiogram; reviewed by 3 neurointerventional experts	Endovascular treatment of completely embolized saccular aneurysms; stent assistance varied	Broad recurrence cohort
He 2023 (10); 302 patients/312 aneurysms; aneurysm-level	Mean 12.53 months	Hypertension recorded as clinical characteristic; operational BP threshold not reported	Any increase in contrast filling during DSA follow-up versus immediate post-procedure angiography	Coil embolization; coil alone and stent-assisted strategies; RROC recorded	Recurrence cohort/prediction model
Andereggen 2024 (19); 333 patients; patient-level	Reported 34 ± 41 months; range 12–265 months; minimum 12 months	Hypertension recorded as vascular risk factor; operational definition not reported	Aneurysm reperfusion during radiologic follow-up; retreatment recorded separately	Conventional coiling with or without supportive balloon/stent devices	Broad durability outcome; omitted from strict sensitivity
Sheng 2019 (21); 113 paraclinoid aneurysms; aneurysm-level	Median 10 (7–18) months recurrent vs. 8 (3–12) months stable	Hypertension history reported; operational BP threshold not reported	Recurrence classification based on follow-up angiography	Endovascular embolization; stent assistance in a subset; CFD-focused cohort	Location-specific CFD cohort
Yuan 2021 (22); 92 MCA bifurcation aneurysms; aneurysm-level	Median 6 (4–10.75) months recurrent vs. 6 (4–7) months non-recurrent; >3 months required	Hypertension history reported; operational BP threshold not reported	Recurrence on follow-up DSA after initial Raymond I complete occlusion	Coils only; totally embolized MCA bifurcation aneurysms; CFD-focused cohort	Location-specific CFD cohort
Zhang 2018 (11); 238 patients/283 aneurysms; aneurysm-level	At least 3 months; 11.4 ± 7.5 months recanalized vs. 9.4 ± 6.3 months non-recanalized	Hypertension recorded as baseline characteristic; operational BP threshold not reported	Any further filling of aneurysm neck or sac on follow-up compared with initial treatment	Coiling or stent-assisted coiling; packing density and CFD measures evaluated	Recanalization cohort with CFD variables
Won 2024 (12); 399 patients/399 unruptured aneurysms; one-to-one patient/aneurysm unit	49.9 ± 20.4 months; range 24–155 months; MRA every 6 months for 2 years then annually	Hypertension recorded as clinical characteristic; operational BP threshold not reported	Coil-mass compaction or aneurysm revisualization with growth; major and minor recurrence included	Stent-assisted coil embolization only; Enterprise/LVIS/Neuroform stents	Long-term recanalization cohort

**Table 2:** Extractable 2 × 2 hypertension-status data used in primary and sensitivity analyses.

Study	Outcome	HTN events/total	No HTN events/total	Non-events HTN/no HTN	OR (95% CI)	Analysis role
Lee 2021 (16) matched	Cumulative recanalization within 36 months	17/126	18/126	109/108	0.94 (0.46–1.91)	Primary
Huang 2017 (9)	ISA recurrence	77/572	6/86	495/80	2.07 (0.87–4.92)	Primary
He 2023 (10)	IA recurrence	14/140	19/172	126/153	0.89 (0.43–1.86)	Primary
Andereggen 2024 (19)	IA reperfusion	78/158	82/175	80/93	1.11 (0.72–1.70)	Primary/sensitivity
Sheng 2019 (21)	Paraclinoid aneurysm recurrence	9/17	35/96	8/61	1.96 (0.69–5.54)	Primary/sensitivity; CFD cohort
Yuan 2021 (22)	MCA bifurcation aneurysm recurrence	7/12	29/80	5/51	2.46 (0.72–8.46)	Primary/sensitivity; CFD cohort
Zhang 2018 (11)	Recanalization	19/40	117/243	21/126	0.97 (0.50–1.90)	Primary
Won 2024 (12)	Recanalization after stent-assisted coiling	21/166	11/233	145/222	2.92 (1.37–6.24)	Primary/sensitivity
Han 2022 (32)	Repeated recurrence after second embolization	3/17	6/27	14/21	0.75 (0.16–3.51)	Sensitivity only
Lee 2021 (16) full cohort	Cumulative recanalization within 36 months	91/484	38/231	393/193	1.18 (0.78–1.78)	Sensitivity only
Lee 2021 (16) full HTN-control	Uncontrolled vs. controlled hypertension	32/146	59/338	114/279	1.33 (0.82–2.15)	BP-control descriptive
Lee 2021 (16) matched HTN-control	Uncontrolled vs. controlled hypertension	7/57	7/57	50/50	1.00 (0.33–3.06)	BP-control descriptive

HTN, hypertension; IA, intracranial aneurysm; ISA, intracranial saccular aneurysm; MCA, middle cerebral artery; CFD, computational fluid dynamics. The primary model used one comparison per study. Won 2024 contributes 21 recanalization events among 166 patients with hypertension and 11 among 233 without hypertension. Sensitivity and BP-control rows were not combined with the primary model.

### Primary hypertension/high-BP status analysis

Eight studies including 2,442 observations were included in the primary random-effects model. A total of 1,231 observations were in the hypertension/high-BP-status group and 1,211 were in the non-hypertension comparator group. The dataset contained 559 recurrence/recanalization/reperfusion events: 242 in the hypertension group and 317 in the non-hypertension group. In Won 2024, recanalization occurred in 21 of 166 patients with hypertension and 11 of 233 patients without hypertension. The primary pooled association was directionally positive but statistically non-significant (OR 1.36, 95% CI 0.92–2.00; *p* = 0.104; *I*^2^ = 31.4%). The primary forest plot is shown in Figure 2A and the quantitative models are summarized in Table 3.

**Figure 2:**
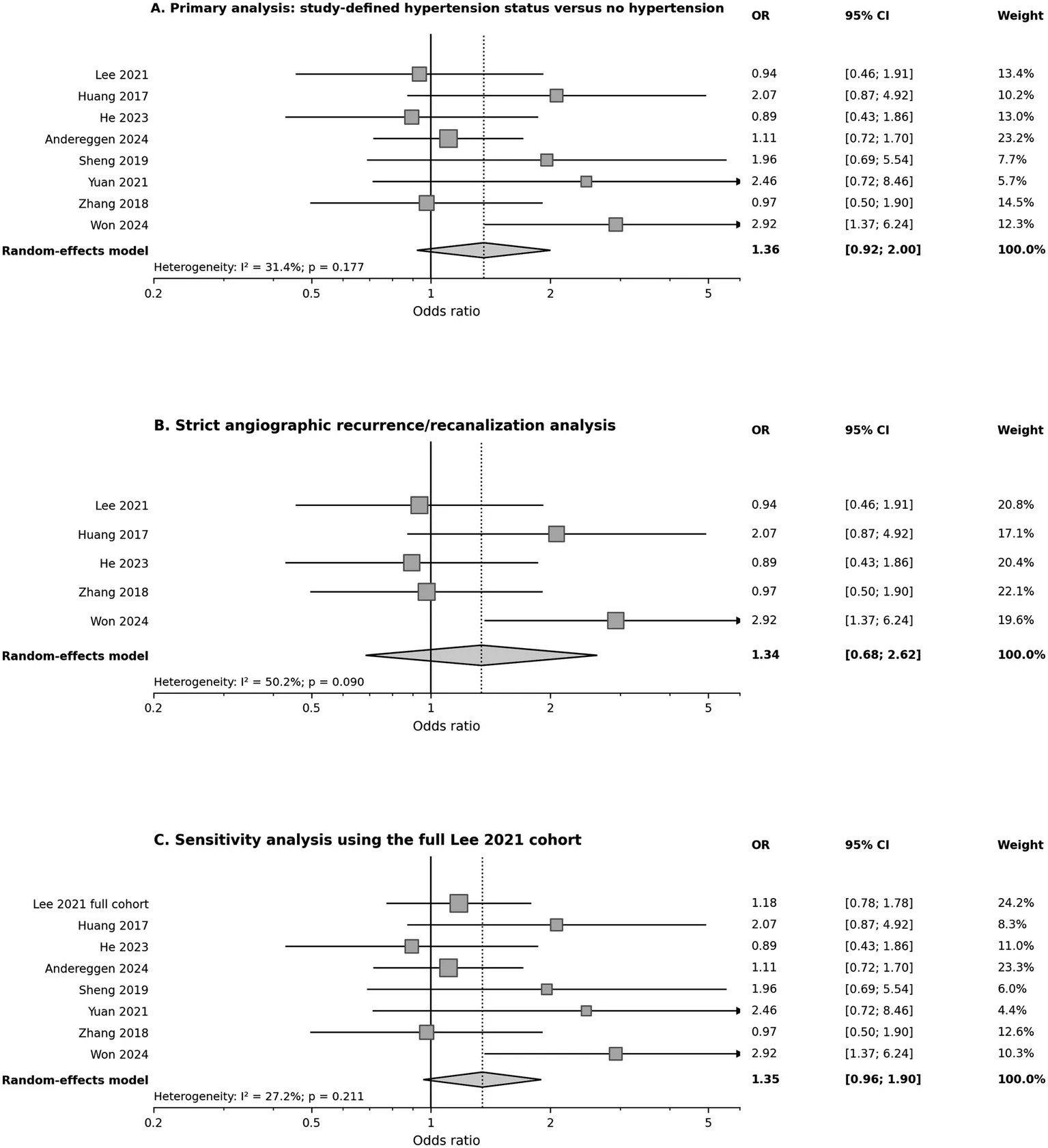
Study-defined hypertension status and recurrence-related outcomes after endovascular treatment. **(A)** Primary broad recurrence-related analysis; **(B)** strict angiographic recurrence/recanalization analysis; **(C)** sensitivity analysis using the full Lee 2021 cohort.

**Table 3:** Summary of direct quantitative and sensitivity analyses.

Analysis	k/source	Effect	95% CI	*p*-value	*I* ^2^	Interpretation
Primary hypertension-status analysis	8	OR 1.36	0.92–2.00	0.104	31.4%	Main direct quantitative analysis
Strict angiographic recurrence/recanalization	5	OR 1.34	0.68–2.62	0.293	50.2%	Omits Andereggen 2024, Sheng 2019, and Yuan 2021
Sensitivity using full Lee 2021 cohort	8	OR 1.35	0.96–1.90	0.079	27.2%	Alternative Lee extraction
Sensitivity including repeated recurrence after second embolization	9	OR 1.32	0.93–1.89	0.108	25.1%	Adds Han 2022
Sensitivity excluding location-specific CFD cohorts	6	OR 1.27	0.78–2.05	0.267	40.5%	Excludes Sheng 2019 and Yuan 2021
Lee 2021 full hypertensive cohort: uncontrolled vs. controlled	Single overlapping estimate	OR 1.33	0.82–2.15	Not pooled	Not applicable	Presented separately
Lee 2021 matched hypertensive cohort: uncontrolled vs. controlled	Single overlapping estimate	OR 1.00	0.33–3.06	Not pooled	Not applicable	Presented separately

### Core sensitivity analyses

Sensitivity analyses did not establish a statistically significant association. The strict angiographic recurrence/recanalization model included five studies and yielded OR 1.34 (95% CI 0.68–2.62; *p* = 0.293; *I*^2^ = 50.2%; Figure 2B). This model omitted Andereggen 2024 because its durability endpoint was the broader construct of aneurysm reperfusion and omitted Sheng 2019 and Yuan 2021 because they were small, location-specific CFD-focused cohorts rather than broader clinical recurrence cohorts. Replacing the matched Lee 2021 cohort with the full Lee 2021 cohort yielded OR 1.35 (95% CI 0.96–1.90; *p* = 0.079; *I*^2^ = 27.2%; Figure 2C). Inclusion of Han 2022, which evaluated repeated recurrence after second embolization, yielded OR 1.32 (95% CI 0.93–1.89; *p* = 0.108; *I*^2^ = 25.1%; Figure 3A). Excluding the location-specific CFD cohorts Sheng 2019 and Yuan 2021 yielded OR 1.27 (95% CI 0.78–2.05; *p* = 0.267; *I*^2^ = 40.5%; Figure 3B).

**Figure 3:**
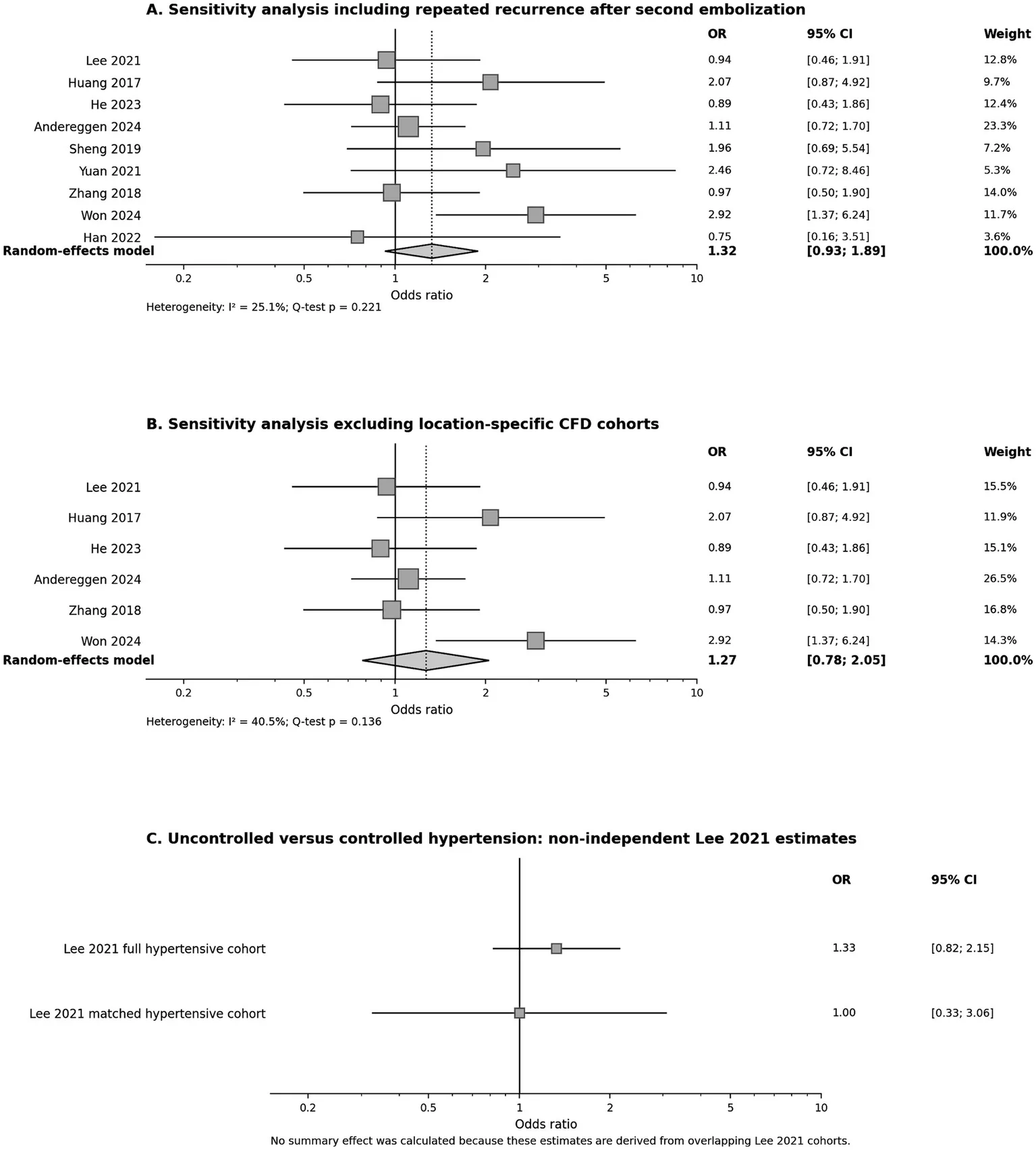
Additional sensitivity and BP-control analyses. **(A)** Sensitivity analysis including repeated recurrence after second embolization; **(B)** sensitivity analysis excluding location-specific CFD cohorts; **(C)** uncontrolled-versus-controlled hypertension estimates from the Lee 2021 full hypertensive cohort and propensity-matched hypertensive cohort. The two panel C estimates arise from overlapping participants and are presented separately without a pooled summary effect.

### Exploratory BP-control and supportive effect-estimate analyses

Controlled-versus-uncontrolled hypertension evidence was confined to Lee 2021. Hypertension was defined by ongoing treatment for diagnosed hypertension or post-coiling SBP > 140 mmHg or DBP > 90 mmHg on more than two consecutive outpatient visits; hypertensive patients were then classified as controlled or uncontrolled using the same BP criteria. Because the full and propensity-matched cohorts overlap, their estimates are presented separately without a combined summary: full hypertensive cohort OR 1.33 (95% CI 0.82–2.15) and matched hypertensive cohort OR 1.00 (95% CI 0.33–3.06; Figure 3C; Table 3). These comparisons do not establish a recurrence-preventive BP target. Figure 4 presents the reported hypertension-related effect estimates from Wiśniewski 2021, Fujimura 2018, and Won 2024 individually, without a pooled summary, because their outcomes and adjustment strategies differ across studies.

**Figure 4:**
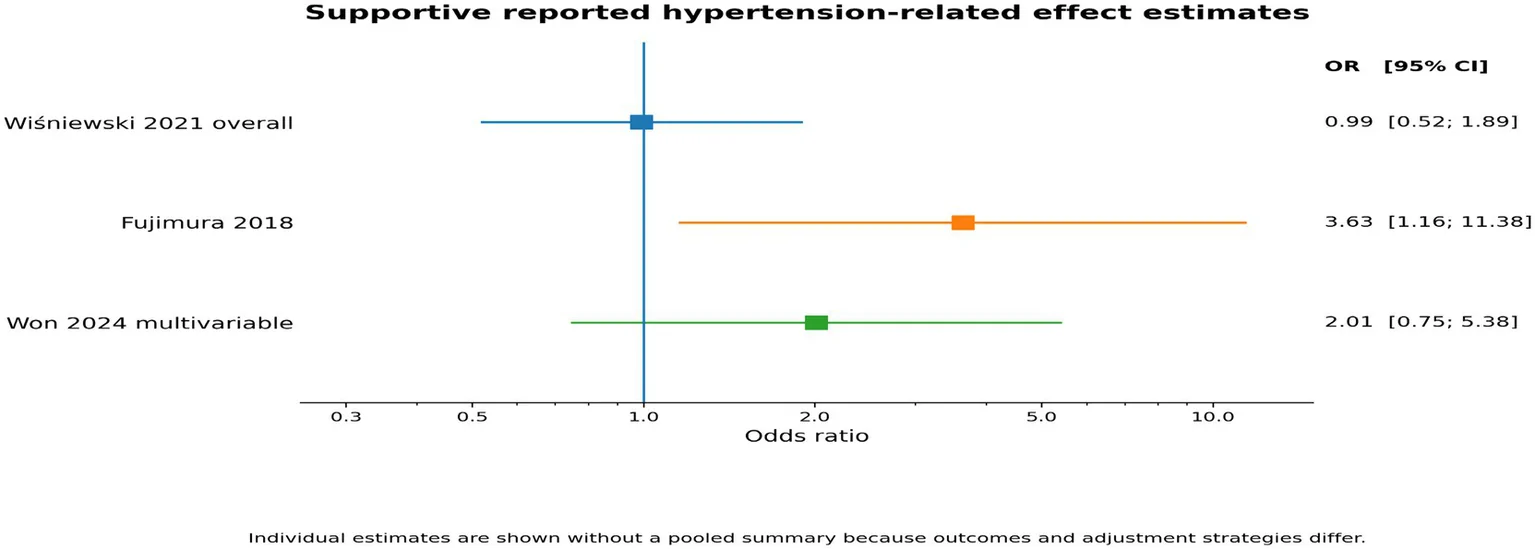
Individual supportive hypertension-related effect estimates from Wiśniewski 2021, Fujimura 2018, and the multivariable Won 2024 model. No pooled summary is calculated because the studies differ in outcome definitions and adjustment strategies. Complementary achieved-BP and BP-variability studies are presented individually in Table 4 and are not pooled because their exposure metrics and clinical outcomes are heterogeneous and not recurrence-specific.

### Target BP values, BP variability, and clinical outcomes

No eligible recurrence study reported angiographic recurrence or recanalization according to achieved post-treatment SBP or DBP target categories. Consequently, no recurrence-preventive BP target can be estimated from the available evidence. Six studies provided complementary clinical evidence on achieved BP or BP variability, but they evaluated functional outcome, mortality, acute rebleeding, or composite vascular prognosis rather than angiographic recurrence. Because the exposure metrics, units, measurement periods, outcomes, and effect measures were heterogeneous and several studies contributed multiple estimates, no pooled BP-variability effect was calculated. Individual findings are reported in Table 4.

**Table 4:** Individual achieved-BP and BP-variability findings from complementary clinical studies; no pooled summary effect was calculated.

Study	BP exposure/metric	Unit or comparison	Measurement period	Clinical outcome	Effect measure	Estimate (95% CI)	Adjustment status
Kim 2021 (17)	Longitudinal SBP	Per 10-mmHg increase	Repeated outpatient BP after aneurysm treatment	Stroke/MI/death composite	HR	1.16 (1.01–1.35)	Adjusted multivariable time-dependent Cox model
Kim 2021 (17)	Longitudinal DBP	Per 10-mmHg increase	Repeated outpatient BP after aneurysm treatment	Stroke/MI/death composite	HR	1.32 (1.06–1.64)	Adjusted multivariable time-dependent Cox model
Ascanio 2020 (33)	Minimum SBP	Per 1-mmHg increase	First 24 h of admission; BP sampled at 4-h intervals	Good functional outcome (mRS 0–2) at last follow-up	OR	1.03 (1.001–1.064)	Adjusted logistic regression
Xu/Bin 2017 (34)	SBP variability, successive variation	Per reported SBPV-SV increment	First 24 h after coil embolization; BP every 2 h	Functional recovery at discharge (mRS/GCS)	OR	0.93 (0.88–0.98)	Adjusted multivariable analysis
Cai 2017 (35)	Mean SBP	120–140 mmHg vs. other mean-SBP categories	Acute post-embolization BP profile	Favorable discharge outcome	OR	7.10 (2.0–25.4)	Adjusted stepwise multivariable logistic analysis
Cai 2017 (35)	SBP variability, successive variation	Per reported SBPV-SV increment	Acute post-embolization BP profile	Functional recovery	OR	0.87 (0.78–0.97)	Adjusted multivariable logistic analysis
Ge 2021 (36)	24-h SBP standard deviation (24hSSD)	≥15 vs. < 15 mmHg	First 24 h after endovascular treatment	Poor 3-month outcome	OR	14.87 (4.00–55.24)	Adjusted multivariable logistic analysis
Lin 2016 (37)	SBP variability, standard deviation	Per reported SD-SBP increment	Hourly before aneurysm repair/censoring in acute <3-day period	Acute aneurysmal rebleeding	OR	1.254 (1.131–1.391)	Binary logistic regression; pre-repair outcome
Lin 2016 (37)	SBP variability, successive variation	Per reported SV-SBP increment	Hourly before aneurysm repair/censoring in acute <3-day period	Acute aneurysmal rebleeding	OR	1.131 (1.039–1.231)	Binary logistic regression; pre-repair outcome

### Hemodynamic and recurrence-predictor evidence

CFD and related hemodynamic studies were retained only as mechanistic evidence. Across these studies, recurrence-related findings involved wall shear stress, flow velocity, pressure difference at the coil surface, residual flow volume, parent-artery geometry, packing/residual volume, and incomplete occlusion. These variables describe local post-treatment hemodynamics and cannot be interpreted as proxies for achieved systemic BP or used to define a systemic recurrence-preventive BP target (Table 5).

**Table 5:** Hemodynamic and recurrence-predictor evidence supporting biological plausibility.

Predictor domain	Representative studies	Interpretation
High WSS at remnant/treatment neck	Luo 2011 (26); Li 2012 (27); Sheng 2019 (21); Yuan 2021 (22)	May promote persistent flow, delayed thrombosis, or local remodeling
High flow velocity/inadequate velocity reduction	Luo 2011 (26); Liu 2016 (25); Zhang 2018 (11); Sheng 2019 (21)	Suggests persistent inflow and unstable occlusion
Pressure difference at coil surface	Nambu 2019 (28); Uno 2021 (29)	Strong CFD-derived recurrence predictor in ICA aneurysm cohorts
Residual flow volume	Umeda 2017 (31)	Quantifies persistent flow after simulated coiling
Parent-artery curvature/geometry	Won 2024 (12)	Geometry may modify post-stent and post-coiling durability
Aneurysm volume, residual volume, packing density	Sadato 2016 (13); Tian 2020 (14); ARETA 2022	Established non-BP predictors that may confound crude hypertension associations

### Leave-one-out analysis and funnel-plot assessment

Leave-one-out analysis did not identify a single study whose removal reversed the direction of the primary association. Omitting Won 2024, the most positive individual study, reduced the pooled estimate and eliminated heterogeneity, suggesting that this cohort contributed substantially to the magnitude of the positive effect. Funnel plots are presented descriptively because all main quantitative models included fewer than ten studies. Funnel plots are shown in Supplementary Figure S1 and leave-one-out analyses in Supplementary Figure S2.

### Risk-of-bias assessment

Domain-level QUIPS assessment indicated predominantly moderate risk of bias in the direct recurrence evidence, driven by retrospective selection, history-based or incompletely defined hypertension exposure, residual confounding, variable imaging follow-up, and reliance on crude 2 × 2 associations. Smaller location-specific CFD cohorts had higher risk of bias for the systemic hypertension question because hypertension was secondary to the mechanistic objective and adjustment was limited. The prospective multicenter ARETA cohort provided strong recurrence-predictor context but did not contribute extractable BP data. Overall confidence in the hypertension-recurrence association is limited, and evidence for a specific recurrence-preventive BP target is especially limited because no eligible recurrence study linked achieved BP target categories to recurrence.

## Discussion

The primary meta-analysis found a directionally higher but statistically non-significant association between study-defined/history-based hypertension and recurrence-related outcomes after endovascular aneurysm treatment (OR 1.36, 95% CI 0.92–2.00). This estimate is derived largely from crude or minimally adjusted observational data and should not be interpreted as an independent causal effect. In Won 2024, the exposure-oriented 2 × 2 table comprises 21/166 recanalization events in patients with hypertension and 11/233 in patients without hypertension; the corresponding study OR remains 2.92 (95% CI 1.37–6.24).

The direct evidence is clinically heterogeneous. Recurrence, recanalization, and reperfusion were not uniformly defined; imaging modalities and follow-up intervals differed; treatment ranged from coiling alone to stent-assisted or device-supported procedures; hypertension was commonly history-based and often lacked an explicit operational threshold; and the analytical unit varied between patients and aneurysms. The strict angiographic sensitivity analysis reduced outcome heterogeneity but retained substantial uncertainty. These differences generate indirectness when a single pooled estimate is interpreted as representing a uniform post-treatment recurrence process.

Confounding is substantial. Aneurysm size, neck width, rupture status, initial occlusion grade, packing density, residual aneurysm volume, parent-artery geometry, and treatment technique are established or repeatedly reported determinants of recurrence (3, 8–15). Several primary studies contributed only crude hypertension-by-outcome counts, so sensitivity analyses can test robustness to study inclusion but cannot eliminate confounding. Accordingly, the evidence does not establish hypertension as an independent recurrence predictor.

The CFD literature supports biological plausibility but remains mechanistically indirect. Persistent high wall shear stress, inadequate velocity reduction, pressure gradients at the coil surface, and residual flow volume may promote reopening or recanalization (11, 21–31). These local variables depend on aneurysm geometry, occlusion, device configuration, and flow boundary assumptions. They complement, rather than validate, the systemic hypertension association and should not be used to infer a specific systemic BP threshold.

Evidence on achieved BP and BP variability is indirect for recurrence. Kim (38) related longitudinal SBP/DBP to a composite vascular prognosis; Ascanio 2020, Xu/Bin 2017, Cai 2017, and Ge 2021 evaluated functional outcomes; and Lin 2016 evaluated acute pre-repair rebleeding. Their exposure metrics, scales, timing, and outcomes differ materially. These individual findings are clinically relevant to broader aneurysm and aSAH management but do not constitute evidence that any achieved SBP/DBP target prevents angiographic recurrence after endovascular treatment.

The evidence gap has direct clinical implications. Blood pressure management remains appropriate for established cardiovascular and cerebrovascular indications and for phase-specific aSAH care, but the recurrence literature does not support prescribing a particular long-term SBP/DBP target specifically to prevent post-endovascular aneurysm recurrence. Prospective studies should record baseline hypertension, antihypertensive therapy, longitudinal achieved BP, visit-to-visit variability, imaging-defined recurrence, analytical unit, and major aneurysm/treatment confounders within the same cohort.

Strengths of this review include separation of direct recurrence evidence from indirect clinical BP and mechanistic CFD evidence, source-level verification of quantitative extractions, multiple clinically motivated sensitivity analyses, and domain-level risk-of-bias interpretation. Limitations include predominantly retrospective designs, heterogeneous recurrence/recanalization/reperfusion definitions, variable endovascular techniques and follow-up, inconsistent hypertension definitions, mixed patient-versus-aneurysm analytical units, limited confounder adjustment, small quantitative models, and the complete absence of recurrence outcomes stratified by achieved SBP/DBP target categories.

## Conclusion

Current evidence is insufficient to establish study-defined hypertension as an independent predictor of recurrence after endovascular treatment of intracranial aneurysms or to recommend a recurrence-preventive blood pressure target. The direct meta-analysis evaluates predominantly history-based hypertension status, not achieved post-treatment BP. Future prospective recurrence studies should integrate longitudinal BP exposure and BP variability with standardized angiographic follow-up and adjustment for aneurysm morphology, occlusion quality, treatment technique, and local hemodynamics.

## Data Availability

The original contributions presented in the study are included in the article/Supplementary material, further inquiries can be directed to the corresponding author.
