# Supplementary material for: Blood pressure status and recurrence risk after endovascular treatment of intracranial aneurysms: a systematic review and meta-analysis with narrative synthesis of blood pressure targets and hemodynamic evidence
Source: Front Neurol. 2026 Oct 26;17:1928054. doi: 10.3389/fneur.2026.1928054 (PMC13745578; doi:10.3389/fneur.2026.1928054)
Supplement: Supplementary file 1 [file Table_1.docx]

**Supplementary Figure S1. Funnel plots**

**Supplementary Figure S1A:** Funnel plot for the primary study-defined hypertension-status analysis

**Supplementary Figure S1B:** Funnel plot for the strict angiographic recurrence/recanalization analysis

**Supplementary Figure S1C:** Funnel plot for the full Lee cohort sensitivity analysis

**Supplementary Figure S1D:** Funnel plot for the repeated-recurrence sensitivity analysis

**Supplementary Figure S1E:** Funnel plot after excluding location-specific CFD cohorts
