# Supplementary material for: Blood pressure status and recurrence risk after endovascular treatment of intracranial aneurysms: a systematic review and meta-analysis with narrative synthesis of blood pressure targets and hemodynamic evidence
Source: Front Neurol. 2026 Oct 26;17:1928054. doi: 10.3389/fneur.2026.1928054 (PMC13745578; doi:10.3389/fneur.2026.1928054)
Supplement: Supplementary file 2 [file Table_2.docx]

**Supplementary Figure S2. Leave-one-out analyses**

**Supplementary Figure S2A:** Leave-one-out analysis for the primary study-defined hypertension-status model

**Supplementary Figure S2B:** Leave-one-out analysis for the strict angiographic recurrence/recanalization model

**Supplementary Figure S2C:** Leave-one-out analysis using the full Lee cohort

**Supplementary Figure S2D:** Leave-one-out analysis including repeated recurrence after second embolization

**Supplementary Figure S2E:** Leave-one-out analysis excluding location-specific CFD cohorts
