# Supplementary material for: Blood pressure status and recurrence risk after endovascular treatment of intracranial aneurysms: a systematic review and meta-analysis with narrative synthesis of blood pressure targets and hemodynamic evidence
Source: Front Neurol. 2026 Oct 26;17:1928054. doi: 10.3389/fneur.2026.1928054 (PMC13745578; doi:10.3389/fneur.2026.1928054)
Supplement: Supplementary file 3 [file Table_3.docx]

**Supplementary Table S1. Search Strategy**

**PubMed/MEDLINE (PubMed)**

| **Coverage** | Database inception through April 2026 |
| --- | --- |
| **Purpose** | Primary BP/hypertension recurrence search |
| **Limits/filters** | Humans; no language or date restriction |
| **Search strategy** | (  "Intracranial Aneurysm"[MeSH Terms]  OR "intracranial aneurysm*"[Title/Abstract]  OR "cerebral aneurysm*"[Title/Abstract]  OR "brain aneurysm*"[Title/Abstract]  OR "ruptured intracranial aneurysm*"[Title/Abstract]  OR "unruptured intracranial aneurysm*"[Title/Abstract]  OR "aneurysmal subarachnoid hemorrhage"[Title/Abstract]  OR "aSAH"[Title/Abstract] ) AND (  "Embolization, Therapeutic"[MeSH Terms]  OR "Endovascular Procedures"[MeSH Terms]  OR "endovascular treatment"[Title/Abstract]  OR "endovascular therapy"[Title/Abstract]  OR "endovascular embolization"[Title/Abstract]  OR "coil embolization"[Title/Abstract]  OR "coiling"[Title/Abstract]  OR "stent-assisted coiling"[Title/Abstract]  OR "balloon-assisted coiling"[Title/Abstract]  OR "flow diverter*"[Title/Abstract]  OR "flow diversion"[Title/Abstract] ) AND (  "Recurrence"[MeSH Terms]  OR recurrence[Title/Abstract]  OR recurrent[Title/Abstract]  OR recanalization[Title/Abstract]  OR recanalisation[Title/Abstract]  OR reperfusion[Title/Abstract]  OR "aneurysm reopening"[Title/Abstract]  OR "aneurysm occlusion"[Title/Abstract]  OR "occlusion status"[Title/Abstract]  OR "Raymond-Roy"[Title/Abstract]  OR "Raymond Roy"[Title/Abstract]  OR retreatment[Title/Abstract]  OR "re-treatment"[Title/Abstract]  OR "repeat embolization"[Title/Abstract] ) AND (  "Hypertension"[MeSH Terms]  OR "Blood Pressure"[MeSH Terms]  OR "Blood Pressure Determination"[MeSH Terms]  OR hypertension[Title/Abstract]  OR hypertensive[Title/Abstract]  OR "high blood pressure"[Title/Abstract]  OR "blood pressure"[Title/Abstract]  OR "systolic blood pressure"[Title/Abstract]  OR "diastolic blood pressure"[Title/Abstract]  OR SBP[Title/Abstract]  OR DBP[Title/Abstract]  OR "mean arterial pressure"[Title/Abstract]  OR MAP[Title/Abstract]  OR "blood pressure control"[Title/Abstract]  OR "blood pressure target*"[Title/Abstract]  OR "target blood pressure"[Title/Abstract]  OR "controlled hypertension"[Title/Abstract]  OR "uncontrolled hypertension"[Title/Abstract]  OR "blood pressure variability"[Title/Abstract]  OR "BP variability"[Title/Abstract]  OR "visit-to-visit variability"[Title/Abstract] ) NOT (  animals[MeSH Terms] NOT humans[MeSH Terms] ) |

**Ovid MEDLINE (Ovid)**

| **Coverage** | Database inception through April 2026 |
| --- | --- |
| **Purpose** | Ovid syntax equivalent of the PubMed strategy |
| **Limits/filters** | Humans; no language or date restriction |
| **Search strategy** | 1. exp Intracranial Aneurysm/ 2. (intracranial aneurysm* or cerebral aneurysm* or brain aneurysm* or ruptured intracranial aneurysm* or unruptured intracranial aneurysm*).tw,kf. 3. (aneurysmal subarachnoid hemorrhage or aneurysmal subarachnoid haemorrhage or aSAH).tw,kf. 4. 1 or 2 or 3  5. exp Embolization, Therapeutic/ 6. exp Endovascular Procedures/ 7. (endovascular treatment or endovascular therapy or endovascular procedure* or endovascular emboli?ation).tw,kf. 8. (coil emboli?ation or coiling or endovascular coiling or detachable coil* or Guglielmi detachable coil*).tw,kf. 9. (stent-assisted coiling or stent assisted coiling or balloon-assisted coiling or balloon assisted coiling).tw,kf. 10. (flow diverter* or flow diversion or pipeline emboli?ation device).tw,kf. 11. 5 or 6 or 7 or 8 or 9 or 10  12. exp Recurrence/ 13. (recurrence or recurrent or recanalization or recanalisation or reperfusion or reopening).tw,kf. 14. (aneurysm reopening or aneurysm recurrence or aneurysm recanalization or aneurysm recanalisation).tw,kf. 15. (retreatment or re-treatment or repeat emboli?ation or re-embolization or re-embolisation).tw,kf. 16. (occlusion status or occlusion worsening or Raymond-Roy or Raymond Roy or Raymond classification).tw,kf. 17. (follow-up angiograph* or angiographic follow-up or DSA follow-up or MRA follow-up).tw,kf. 18. 12 or 13 or 14 or 15 or 16 or 17  19. exp Hypertension/ 20. exp Blood Pressure/ 21. exp Blood Pressure Determination/ 22. (hypertension or hypertensive or high blood pressure or elevated blood pressure).tw,kf. 23. (blood pressure or systolic blood pressure or diastolic blood pressure or mean arterial pressure).tw,kf. 24. (SBP or DBP or MAP).tw,kf. 25. (blood pressure control or BP control or controlled hypertension or uncontrolled hypertension).tw,kf. 26. (blood pressure target* or target blood pressure or BP target*).tw,kf. 27. (blood pressure variab* or BP variab* or systolic blood pressure variab* or diastolic blood pressure variab*).tw,kf. 28. (visit-to-visit variab* or successive variation or standard deviation or coefficient of variation).tw,kf. 29. 19 or 20 or 21 or 22 or 23 or 24 or 25 or 26 or 27 or 28  30. 4 and 11 and 18 and 29  31. animals/ not humans/ 32. 30 not 31 |

**Embase**

| **Coverage** | Database inception through April 2026 |
| --- | --- |
| **Purpose** | Embase Emtree/free-text strategy |
| **Limits/filters** | Humans; no language or date restriction |
| **Search strategy** | 1. exp intracranial aneurysm/ 2. (intracranial aneurysm* or cerebral aneurysm* or brain aneurysm*).tw,kw. 3. (aneurysmal subarachnoid hemorrhage or aneurysmal subarachnoid haemorrhage or aSAH).tw,kw. 4. 1 or 2 or 3  5. exp endovascular surgery/ 6. exp embolization/ 7. (endovascular treatment or endovascular therapy or endovascular procedure* or endovascular emboli?ation).tw,kw. 8. (coil emboli?ation or coiling or detachable coil* or stent-assisted coiling or balloon-assisted coiling).tw,kw. 9. (flow diverter* or flow diversion or pipeline emboli?ation device).tw,kw. 10. 5 or 6 or 7 or 8 or 9  11. exp recurrent disease/ 12. (recurrence or recurrent or recanalization or recanalisation or reperfusion or reopening).tw,kw. 13. (retreatment or re-treatment or repeat emboli?ation or re-embolization or re-embolisation).tw,kw. 14. (occlusion status or Raymond-Roy or Raymond Roy or angiographic follow-up or follow-up angiograph*).tw,kw. 15. 11 or 12 or 13 or 14  16. exp hypertension/ 17. exp blood pressure/ 18. (hypertension or hypertensive or high blood pressure or elevated blood pressure).tw,kw. 19. (blood pressure or systolic blood pressure or diastolic blood pressure or mean arterial pressure).tw,kw. 20. (SBP or DBP or MAP).tw,kw. 21. (blood pressure control or BP control or controlled hypertension or uncontrolled hypertension).tw,kw. 22. (blood pressure target* or target blood pressure or BP target*).tw,kw. 23. (blood pressure variab* or BP variab* or visit-to-visit variab* or successive variation or coefficient of variation).tw,kw. 24. 16 or 17 or 18 or 19 or 20 or 21 or 22 or 23  25. 4 and 10 and 15 and 24  26. animal/ not human/ 27. 25 not 26 |

**PubMed/MEDLINE supplementary (PubMed)**

| **Coverage** | Database inception through April 2026 |
| --- | --- |
| **Purpose** | Broader recurrence search without mandatory BP terms |
| **Limits/filters** | Humans; no language or date restriction |
| **Search strategy** | Supplementary broader recurrence search without mandatory BP terms: (  "Intracranial Aneurysm"[MeSH Terms]  OR "intracranial aneurysm*"[Title/Abstract]  OR "cerebral aneurysm*"[Title/Abstract]  OR "aneurysmal subarachnoid hemorrhage"[Title/Abstract]  OR "aSAH"[Title/Abstract] ) AND (  "Embolization, Therapeutic"[MeSH Terms]  OR "Endovascular Procedures"[MeSH Terms]  OR embolization[Title/Abstract]  OR embolisation[Title/Abstract]  OR endovascular[Title/Abstract]  OR coiling[Title/Abstract]  OR "flow diversion"[Title/Abstract] ) AND (  recurrence[Title/Abstract]  OR recurrent[Title/Abstract]  OR recanalization[Title/Abstract]  OR recanalisation[Title/Abstract]  OR reperfusion[Title/Abstract]  OR retreatment[Title/Abstract]  OR "occlusion status"[Title/Abstract]  OR "angiographic follow-up"[Title/Abstract] ) |
