# Supplementary material for: Blood pressure status and recurrence risk after endovascular treatment of intracranial aneurysms: a systematic review and meta-analysis with narrative synthesis of blood pressure targets and hemodynamic evidence
Source: Front Neurol. 2026 Oct 26;17:1928054. doi: 10.3389/fneur.2026.1928054 (PMC13745578; doi:10.3389/fneur.2026.1928054)
Supplement: Supplementary file 4 [file Table_4.docx]

Supplementary Table S2. QUIPS domain-level risk-of-bias assessment of included studies

Risk of bias was assessed independently by two reviewers using the Quality In Prognosis Studies (QUIPS) framework. Disagreements were resolved through discussion and consensus. Domains were study participation, study attrition, prognostic-factor measurement, outcome measurement, study confounding, and statistical analysis/reporting. Each domain and the overall study were judged as low, moderate, or high risk of bias.

| **No.** | **Study** | **Study participation** | **Study attrition** | **Prognostic-factor measurement** | **Outcome measurement** | **Study confounding** | **Statistical analysis/reporting** | **Overall RoB** | **Justification** |
| --- | --- | --- | --- | --- | --- | --- | --- | --- | --- |
| 1 | Lee 2021 | Moderate | Moderate | Low | Low | Moderate | Low | Moderate | Direct BP-focused recurrence study with hypertension and controlled/uncontrolled hypertension definitions; residual confounding and retrospective follow-up remain possible. |
| 2 | Huang 2017 | Moderate | Moderate | Moderate | Low | Moderate | Low | Moderate | Large recurrence cohort with extractable hypertension data; hypertension based on clinical history and residual confounding by morphology/treatment factors likely. |
| 3 | He 2023 | Moderate | Moderate | Moderate | Low | Moderate | Low | Moderate | Predictive model study with recurrence data; independent predictors focused on aneurysm/treatment factors rather than BP; retrospective confounding remains. |
| 4 | Andereggen 2024 | Moderate | Moderate | Moderate | Low | Moderate | Low | Moderate | Long-term outcome dataset with hypertension and reperfusion information; outcome definition broader than strict recanalization and BP exposure was not granular. |
| 5 | Sheng 2019 | Moderate | Moderate | Moderate | Low | High | Moderate | High | Useful HTN counts and hemodynamic data, but systemic BP association is unadjusted and cohort is location-specific with CFD assumptions. |
| 6 | Yuan 2021 | Moderate | Moderate | Moderate | Low | High | Moderate | High | Location-specific total-embolization CFD cohort; hypertension data are secondary and unadjusted for the BP question. |
| 7 | Zhang 2018 | Moderate | Moderate | Moderate | Low | Moderate | Low | Moderate | Multivariate recurrence analysis with extractable HTN counts and hemodynamic predictors; BP exposure remains history-based. |
| 8 | Won 2024 | Moderate | Moderate | Low | Low | Moderate | Low | Moderate | Long-term stent-assisted coiling follow-up with multivariable modeling; hypertension significant only in univariate analysis. |
| 9 | Han 2022 | Moderate | Moderate | Moderate | Low | High | Moderate | High | Small and selected second-embolization cohort; suitable only for sensitivity because it does not represent initial post-embolization recurrence. |
| 10 | Wiśniewski 2021 | Moderate | Moderate | Moderate | Low | Moderate | Moderate | Moderate | Provides reported hypertension OR and CFD evidence but no extracted raw 2x2 BP-status data for the main model. |
| 11 | Fujimura 2018 | Moderate | Moderate | Moderate | Low | Moderate | Low | Moderate | Includes adjusted hypertension-related effect for retreatment due to recanalization; outcome and exposure differ from primary recurrence model. |
| 12 | Kim 2021 | Low | Moderate | Low | Moderate | Moderate | Low | Moderate | Large longitudinal BP prognosis study; outcome is composite clinical prognosis and includes clipping, so indirect for recurrence. |
| 13 | Ascanio 2020 | Moderate | Moderate | Low | Moderate | Moderate | Moderate | Moderate | Evaluates acute BP variability and functional outcome; retrospective clinical-outcome design with indirect relevance to recurrence. |
| 14 | Xu/Bin 2017 | Moderate | Moderate | Low | Moderate | Moderate | Low | Moderate | Direct post-coiling BP variability measurement but outcome is discharge clinical status rather than angiographic recurrence. |
| 15 | Cai 2017 | Moderate | Moderate | Low | Moderate | Moderate | Low | Moderate | Provides indirect target SBP evidence (120-140 mmHg) for clinical outcome; not recurrence-stratified. |
| 16 | Ge 2021 | Moderate | Moderate | Low | Low | Moderate | Moderate | Moderate | BP variability predicts 3-month clinical outcome after endovascular treatment; not an angiographic recurrence study. |
| 17 | Lin 2016 | Moderate | Moderate | Low | Low | Moderate | Low | Moderate | Relevant to BP variability and rebleeding risk; outcome precedes definitive post-embolization recurrence assessment. |
| 18 | Liu 2016 | Moderate | Moderate | Low | Moderate | High | Moderate | High | Small location-specific CFD study; valuable mechanistic evidence but high confounding risk for causal inference. |
| 19 | Luo 2011 | High | Moderate | Moderate | Low | High | Moderate | High | Very small sample; CFD assumptions and selection limit confidence despite recurrence-relevant mechanistic information. |
| 20 | Li 2012 | High | Moderate | Moderate | Low | High | Moderate | High | Small CFD sample and modeling assumptions; useful as mechanistic evidence rather than definitive predictor evidence. |
| 21 | Nambu 2019 | Moderate | Moderate | Low | Low | Moderate | Low | Moderate | Moderate-size ICA aneurysm CFD cohort with pressure-difference predictor; limited by retrospective and location-specific design. |
| 22 | Uno 2021 | Moderate | Moderate | Low | Low | Moderate | Low | Moderate | Supports pressure-difference predictor with real post-coiling geometry; still retrospective and location-specific. |
| 23 | Liao 2024 | Moderate | Moderate | Low | Low | Moderate | Low | Moderate | Uses hemodynamic modeling to predict recanalization; indirect to systemic BP and limited by model assumptions. |
| 24 | Umeda 2017 | Moderate | Moderate | Low | Low | Moderate | Low | Moderate | Residual flow volume independently predicted recurrence; limited by small sample and CFD modeling assumptions. |
| 25 | Sadato 2016 | Moderate | Moderate | Low | Low | Moderate | Low | Moderate | Large morphologic/packing dataset showing residual volume association with recanalization; no systemic BP exposure. |
| 26 | Nambu 2022 | Moderate | Moderate | Moderate | Low | Moderate | Low | Moderate | Clinically useful recurrence-pattern evidence; not designed for BP exposure or target BP assessment. |
| 27 | Pierot/ARETA 2022 | Low | Moderate | Low | Low | Low | Low | Moderate | High-quality prospective multicenter recurrence-predictor study; no extractable BP data but strong contextual evidence. |
| 28 | Tian 2020 | Moderate | Moderate | Low | Low | Moderate | Low | Moderate | Multicenter recurrence cohort identifying packing density; isolated hypertension data not extractable for main model. |
| 29 | Ries 2007 | High | High | Low | Low | Moderate | Moderate | High | Long follow-up retreatment evidence, but substantial loss to follow-up and older retrospective design increase bias. |
| 30 | Tomalski 2020 | Moderate | Moderate | Moderate | Low | Moderate | Moderate | Moderate | Evaluated hypertension among covariates but reported only aneurysm size as independent factor; limited extractability. |
| 31 | Repić Buličić 2024 | Moderate | Moderate | Moderate | Low | Moderate | Moderate | Moderate | Provides contemporary clinical experience and retreatment data; no hypertension-recurrence table. |
