# Supplementary material for: Blood pressure status and recurrence risk after endovascular treatment of intracranial aneurysms: a systematic review and meta-analysis with narrative synthesis of blood pressure targets and hemodynamic evidence
Source: Front Neurol. 2026 Oct 26;17:1928054. doi: 10.3389/fneur.2026.1928054 (PMC13745578; doi:10.3389/fneur.2026.1928054)
Supplement: Supplementary file 5 [file Table_5.docx]

**Supplementary Table S3. Study-level risk-of-bias interpretation**

Overall judgments synthesize the QUIPS domain-level assessment in Supplementary Table S2.

| **No.** | **Study** | **Role in current study** | **Overall risk of bias** | **Principal limitations** |
| --- | --- | --- | --- | --- |
| 1 | Lee 2021 | Retrospective matched cohort; primary hypertension/high-BP status meta-analysis | Moderate | Direct BP-focused recurrence study with hypertension and controlled/uncontrolled hypertension definitions; residual confounding and retrospective follow-up remain possible. |
| 2 | Huang 2017 | Retrospective recurrence-predictor cohort; primary meta-analysis | Moderate | Large recurrence cohort with extractable hypertension data; hypertension based on clinical history and residual confounding by morphology/treatment factors likely. |
| 3 | He 2023 | Retrospective cohort / prediction model; primary meta-analysis | Moderate | Predictive model study with recurrence data; independent predictors focused on aneurysm/treatment factors rather than BP; retrospective confounding remains. |
| 4 | Andereggen 2024 | Retrospective long-term coiled aneurysm cohort; primary broad recurrence/reperfusion model | Moderate | Long-term outcome dataset with hypertension and reperfusion information; outcome definition broader than strict recanalization and BP exposure was not granular. |
| 5 | Sheng 2019 | Retrospective location-specific CFD cohort; primary meta-analysis and hemodynamic review | High | Useful HTN counts and hemodynamic data, but systemic BP association is unadjusted and cohort is location-specific with CFD assumptions. |
| 6 | Yuan 2021 | Retrospective MCA bifurcation CFD cohort; primary meta-analysis and hemodynamic review | High | Location-specific total-embolization CFD cohort; hypertension data are secondary and unadjusted for the BP question. |
| 7 | Zhang 2018 | Retrospective multivariate cohort with CFD variables; primary meta-analysis and hemodynamic review | Moderate | Multivariate recurrence analysis with extractable HTN counts and hemodynamic predictors; BP exposure remains history-based. |
| 8 | Won 2024 | Retrospective stent-assisted coiling cohort; primary meta-analysis | Moderate | Long-term stent-assisted coiling follow-up with multivariable modeling; hypertension significant only in univariate analysis. |
| 9 | Han 2022 | Retrospective repeated-recurrence after second embolization cohort; sensitivity analysis only | High | Small and selected second-embolization cohort; suitable only for sensitivity because it does not represent initial post-embolization recurrence. |
| 10 | Wiśniewski 2021 | Retrospective clinical/CFD cohort; supportive reported-OR analysis and hemodynamic review | Moderate | Provides reported hypertension OR and CFD evidence but no extracted raw 2x2 BP-status data for the main model. |
| 11 | Fujimura 2018 | Retrospective CFD multivariable retreatment cohort; supportive reported-OR analysis | Moderate | Includes adjusted hypertension-related effect for retreatment due to recanalization; outcome and exposure differ from primary recurrence model. |
| 12 | Kim 2021 | Nationwide population-based cohort; target BP/BP-control systematic review | Moderate | Large longitudinal BP prognosis study; outcome is composite clinical prognosis and includes clipping, so indirect for recurrence. |
| 13 | Ascanio 2020 | Retrospective aSAH cohort; BP variability systematic review | Moderate | Evaluates acute BP variability and functional outcome; retrospective clinical-outcome design with indirect relevance to recurrence. |
| 14 | Xu/Bin 2017 | Retrospective ruptured aneurysm coiling cohort; BP variability systematic review | Moderate | Direct post-coiling BP variability measurement but outcome is discharge clinical status rather than angiographic recurrence. |
| 15 | Cai 2017 | Retrospective poor-grade aSAH after coiling cohort; target BP/BP variability review | Moderate | Provides indirect target SBP evidence (120-140 mmHg) for clinical outcome; not recurrence-stratified. |
| 16 | Ge 2021 | Retrospective aSAH endovascular cohort; BP variability systematic review | Moderate | BP variability predicts 3-month clinical outcome after endovascular treatment; not an angiographic recurrence study. |
| 17 | Lin 2016 | Case-control aSAH rebleeding study; BP variability/rebleeding systematic review | Moderate | Relevant to BP variability and rebleeding risk; outcome precedes definitive post-embolization recurrence assessment. |
| 18 | Liu 2016 | Retrospective CFD cohort; hemodynamic systematic review | High | Small location-specific CFD study; valuable mechanistic evidence but high confounding risk for causal inference. |
| 19 | Luo 2011 | Small retrospective CFD cohort; hemodynamic systematic review | High | Very small sample; CFD assumptions and selection limit confidence despite recurrence-relevant mechanistic information. |
| 20 | Li 2012 | Small patient-specific CFD simulation cohort; hemodynamic systematic review | High | Small CFD sample and modeling assumptions; useful as mechanistic evidence rather than definitive predictor evidence. |
| 21 | Nambu 2019 | Retrospective CFD cohort; hemodynamic systematic review | Moderate | Moderate-size ICA aneurysm CFD cohort with pressure-difference predictor; limited by retrospective and location-specific design. |
| 22 | Uno 2021 | Retrospective CFD cohort using real post-coiling models; hemodynamic systematic review | Moderate | Supports pressure-difference predictor with real post-coiling geometry; still retrospective and location-specific. |
| 23 | Liao 2024 | Retrospective CFD modeling study; hemodynamic systematic review | Moderate | Uses hemodynamic modeling to predict recanalization; indirect to systemic BP and limited by model assumptions. |
| 24 | Umeda 2017 | Retrospective porous-media CFD cohort; hemodynamic systematic review | Moderate | Residual flow volume independently predicted recurrence; limited by small sample and CFD modeling assumptions. |
| 25 | Sadato 2016 | Retrospective morphologic/packing cohort; recurrence-predictor systematic review | Moderate | Large morphologic/packing dataset showing residual volume association with recanalization; no systemic BP exposure. |
| 26 | Nambu 2022 | Retrospective recurrence-pattern cohort; recurrence-consequence systematic review | Moderate | Clinically useful recurrence-pattern evidence; not designed for BP exposure or target BP assessment. |
| 27 | Pierot/ARETA 2022 | Prospective multicenter cohort; recurrence-predictor systematic review | Moderate | High-quality prospective multicenter recurrence-predictor study; no extractable BP data but strong contextual evidence. |
| 28 | Tian 2020 | Retrospective multicenter recurrence cohort; recurrence-predictor systematic review | Moderate | Multicenter recurrence cohort identifying packing density; isolated hypertension data not extractable for main model. |
| 29 | Ries 2007 | Retrospective long-term retreatment cohort; retreatment-predictor systematic review | High | Long follow-up retreatment evidence, but substantial loss to follow-up and older retrospective design increase bias. |
| 30 | Tomalski 2020 | Retrospective recurrence cohort; recurrence-predictor systematic review | Moderate | Evaluated hypertension among covariates but reported only aneurysm size as independent factor; limited extractability. |
| 31 | Repić Buličić 2024 | Retrospective single-center experience; clinical recurrence/retreatment systematic review | Moderate | Provides contemporary clinical experience and retreatment data; no hypertension-recurrence table. |
